# Capacity for Alzheimer’s Disease confirmatory testing in the US ‐ current situation and simulations for future increase

**DOI:** 10.1002/alz.085540

**Published:** 2025-01-09

**Authors:** Sophie Roth, Jessie T. Yan, Maria‐Magdalena Patru, Soeren Mattke, Gustaf Ortsäter, Anders Gustavsson

**Affiliations:** ^1^ Roche Diagnostics International AG, Rotkreuz, Zug Switzerland; ^2^ Roche Molecular Systems, Inc., Santa Clara, CA USA; ^3^ Roche Diagnostics Corporation, Indianapolis, IN USA; ^4^ University of Southern California, Los Angeles, CA USA; ^5^ Quantify Research, Stockholm, Stockholm Sweden; ^6^ Quantify Research, Stokholm, Stockholm Sweden

## Abstract

**Background:**

Recent advances in Alzheimer’s Disease (AD) disease modifying therapies (DMTs) emphasize the need of an early, timely and accurate diagnosis to initiate treatment and improve patient outcomes. Clinicians must confirm amyloid pathology through cerebrospinal fluid (CSF) testing or amyloid positron emission tomography (PET) scan to qualify patients. As a result, the demand for confirmatory CSF or imaging biomarker testing is predicted to rise significantly.

**Method:**

We developed a model to analyze diagnostic capacity for patients with mild cognitive impairment (MCI) seeking care in the US. We assessed amyloid PET and CSF testing from a payer’s perspective over 5 years. It was assumed that CSF testing is constrained by the number of lumbar puncture procedures rather than laboratory capacity. Of all currently available PET capacity per year, about 5% is used for AD diagnosis; this is assumed to increase approximately 7% per year. We compare four scenarios: 1) currently observed CSF (about 3.5%) and amyloid PET (< 1%) usage based on Medicare data; 2) increasing CSF use to meet the increasing demand for confirmatory testing, while PET remains as in scenario 1; 3) 100% amyloid PET 4) 100% CSF usage.

**Result:**

Current usage of CSF and amyloid PET testing (scenario 1) cannot meet the increasing demand for AD confirmatory testing: Assuming 50% of patients with MCI seek care annually, there would be an excess demand of about 10 times in year 1. Increasing CSF usage in scenario 2 would lead to timely diagnosis of up to 55% of beta amyloid positive patients in 5 years, of which 45% is attributed to CSF testing and the remaining to PET use. The more extreme scenarios 3 and 4 of 100% use of either PET or CSF, result in <1% patients and 12.6% patients being diagnosed in year 1, respectively.

**Conclusion:**

Increasing CSF use and capacity could meet the demand for confirmatory testing the quickest, reduce wait times and increase the number of timely diagnosed patients, especially during the time where more amyloid PET capacity is being instituted. Efforts to increase CSF usage and capacity could positively impact patient outcomes.

